# Patterns of Phenolic Compounds in *Betula* and *Pinus* Pollen

**DOI:** 10.3390/plants12020356

**Published:** 2023-01-12

**Authors:** Ilona Kerienė, Ingrida Šaulienė, Laura Šukienė, Asta Judžentienė, Magdalena Ligor, Bogusław Buszewski

**Affiliations:** 1 Regional Development Institute, Šiauliai Academy, Vilnius University, 84 Vytauto Str., LT-76352 Šiauliai, Lithuania; 2 Center for Physical Sciences and Technology, Department of Organic Chemistry, Saulėtekio Avenue 3, LT-10257 Vilnius, Lithuania; 3 Life Sciences Center, Institute of Biosciences, Vilnius University, Saulėtekio Avenue 7, LT-10257 Vilnius, Lithuania; 4 Department of Environmental Chemistry and Bioanalytics, Faculty of Chemistry, Nicolaus Copernicus University, 7 Gagarina Str., 87-100 Torun, Poland

**Keywords:** *Pinus*, *Betula*, pollen, bound phenolic compounds, free phenolic compounds, bioactivity, phenolic acids, flavonoids

## Abstract

In this study, phenolic compounds and their antioxidant activity in the pollen of anemophilous *Betula* and *Pinus* were determined. Spectrophotometric, high-performance thin-layer and liquid chromatography methods were applied. Free phenolic compounds (free PC) and phenolic compounds bound to the cell wall (bound PC) were analysed in the pollen extracts. Regardless of the pollen species, their content was 20% higher than that in bound PC extracts. *Pinus* pollen extracts contained 2.5 times less phenolic compounds compared to *Betula*. Free PC extraction from the deeper layers of *Pinus* pollen was minimal; the same content of phenolic compounds was obtained in both types of extracts. The bioactivity of pollen (*p* < 0.05) is related to the content of phenolic compounds and flavonoids in *Betula* free PC and in bound PC, and only in free PC extracts of *Pinus*. Rutin, chlorogenic and trans-ferulic acids were characterised by antioxidant activity. Phenolic acids accounted for 70–94%, while rutin constituted 2–3% of the total amount in the extracts. One of the dominant phenolic acids was trans-ferulic acid in all the *Betula* and *Pinus* samples. The specific compounds were vanillic and chlorogenic acids of *Betula* pollen extracts, while *Pinus* extracts contained gallic acid. The data obtained for the phenolic profiles and antioxidant activity of *Betula* and *Pinus* pollen can be useful for modelling food chains in ecosystems.

## 1. Introduction

Pollen is an integral part of sexual reproduction in the ontogeny of gymnosperms and angiosperms. The specific structure of pollen ensures the transportation of the genetic material contained in it [1]. A tough exterior membrane consists of two layers: the exine, exteriorly and the intina adhering to the protoplasm, interiorly. The exine is permeable by water and chemical compounds dissolved in it and is elastic. It contains mechanically resistant and chemically inert biopolymer sporopollenin. Its molecules are bound by phenolic compounds [2,3,4,5]. The intina contains cellulose and pectins [6], which are usually combined with phenolic compounds [7,8]. Free phenolic compounds are synthesised in the cytosol of plant cells [9]. In pollen, phenolic compounds are involved in the regulation of the cell reproduction cycle, they are important for pollen development, male fertility, pollen germination, tube growth [10,11] and protection against the harmful impact of radiation [2,12]. In ecosystems, the importance of pollen is not limited to the transfer of genetic information only. Pollen dispersed during the flowering of anemophilous plants can affect the functioning of food chains [13]. Much evidence has been collected to the effect that pollen entering natural water bodies enriches them with nutrients [14,15,16,17,18]. Heterotrophic bacteria and aquatic microscopic fungi decompose pollen dispersed in water before it becomes part of the trophic chain [18]. The positive effect is multifaceted. For instance, pollen admixtures significantly increase the total biomass of phytoplankton and that of herbivorous zooplankton, resulting in a higher abundance of filamentous green algae and the large diatoms [15]. However, pollen rains are temporary nutrient impulses, they enter food chains quickly and are rapidly utilised; therefore, estimates of annual biomass input can be misleading [19].

Pollen of entomophilous plants collected by bees is more valued in the human diet due to its health-giving properties [20,21] and the antifungal impact of its bioactive compounds [22]. Although pollen of anemophilous plants is characterised by higher purity and more stable components in comparison with entomophilous pollen, it is used less frequently for human needs [23]. In addition, some pollen that is widely dispersed by wind-pollinated plants can affect sensitive people and cause health problems. Allergenic pollen causes allergies in up to 25% of the population worldwide [24].

Anemophilic plants of the genera *Betula* and *Pinus* disperse a particularly large amount of pollen during vegetation. *Pinus* are gymnosperms; therefore, unlike angiosperms (e.g., *Betula*), their pollen is not enclosed in the cotyledons. According to [25], one inflorescence of *Betula verrucosa* contains an average of 900 stamens, each of which matures about 11,160 pollen grains (~10 million pollen grains per inflorescence). Pollen production is variable and varies from year to year. For example, *Betula pendula* pollen production per catkin averaged 8.2 million in 2002 and 4.8 million in 2003 [26]. Forests produce from 100 to 1000 kg/ha of *Pinus* pollen biomass [27], most of which is deposited on the forest floor and in lakes. *Pinus* pollen forms a yellow film when dispersed in bodies of water and can make up to 30–40% of the total of all suspended solids in water [28]. During hydration, the pollen swells, the exine disintegrates, and within a few days, the pollen germinates [29]. According to [30], the sources of the dispersal of substances from pollen can be both the outer surface and the interior of the pollen: upon contact with water, the substances are washed off the surface of the pollen [31,32], or, depending on the size and elasticity of the pollen pore, it ruptures and disperses the contents of the cytoplasm [33].

The biochemical composition of gymnosperm and angiosperm pollen is similar [29,34,35]. The pollen cell wall contains cellulose, hemicellulose, pectin, proteins, long-chain wax esters and phenylpropanoids [34,35,36,37]. There exist differences in the amount of these molecules [34] or they are distributed differently in the pollen cell wall tubes [29]. Pollen of both gymnosperms and angiosperms contains phenolic compounds important for cell development: ferulic acid, p-coumaric acid, hydroxybenzoic acids and sinapic acid [2,5,34,38]. Knowledge of these compounds is ambiguous and depends on research objectives and methods. The methods and extraction solvents used for the extraction of phenolic compounds under laboratory conditions depend greatly on the types of phenolic compounds to be extracted. In order to speed up the extraction process, pollen is grated by mixers [39], and processed by cold [36,40]. In pollen, due to the stability of sporopollenin, the exine is resistant to both alkalis and concentrated inorganic acids [41]. Methods including acetolysis, methanol acid extraction and alkaline hydrolysis are used for the extraction of bioactive phenolic compounds [2,3,5]. Cell wall-bound phenolic compounds are covalently linked to the cell wall matrix by ester, ether and C–C bonds. Their breaking from the cell wall is carried out by alkaline, acidic and enzymatic hydrolysis [42,43]. Since some phenolic compounds (such as chlorogenic acid) lose their stability at low pH [44], water-based alkaline hydrolysis is more commonly used to prepare bound phenolic compounds extracts [42]. The stability and bioactivity of phenolic compounds bound to the cell wall and produced in the cytosol of the cell can be affected by mechanical damage to the pollen wall and irradiation with radioactive substances, for instance, when radioactive 60 Co is used as an irradiation source [8].

Free phenolic compounds produced in the cell cytosol are less soluble in water, thus ethanol and methanol are often used as their extraction solvents [45]. Ultrasound, Soxhlet, supercritical fluid, solid phase extractions and other methods are used for the extraction of free phenolic compounds from various matrices [46]. Ultrasonic extraction is convenient to use because the method is not time consuming and ultrasonic vibrations increase the efficiency of solvent penetration into the sample [47,48,49]. To increase the interaction of the solvent with the surface area of the sample, the samples are suspended in an appropriate volume of solvent and shaken with an orbital shaking device [21].

To our knowledge, phenolic compounds and their bioactive properties in *Betula* and *Pinus* pollen have been studied exceptionally rarely. Based on these assumptions, we defined the methods of isolating free phenolic compounds in pollen, and studied the total content of phenolic substances, antioxidant activity and distribution of individual phenolic compounds in *Betula* and *Pinus* pollen. Information on the pollen phenolic compound profiles and antioxidant activity of the most abundant pollen of anemophilous plants may be useful for modelling food chains in ecosystems, especially in water bodies, or as parameters for assessing trophic potential.

## 2. Results

### 2.1. Application of Different Methods for the Extraction of Free Phenolic Compounds from Betula pendula and Pinus sylvestris Pollen

The results of experiments evaluating the extraction efficiency of phenolic compounds are presented in Table 1. It should be noted that the mechanical processing of pollen with liquid nitrogen and the application of different durations of ultrasonic shaking had no essential effect on the total amount of free PC in *B*. *pendula* and *P. sylvestris* pollen extracts. Mechanical nitrogen treatment did not affect pollen wall disintegration. Regardless of the duration of sample shaking, the average concentration of free PC in *B. pendula* pollen reached 1.09 ± 0.03 mg/mL. During extraction from *P. sylvestris* pollen by this method, 2.5 times fewer (0.38 ± 0.01 mg/mL) phenolic compounds were obtained compared to *B. pendula*. The results show that to isolate phenolic compounds from pollen by ultrasonic method, it is sufficient to shake samples for 10 min using a shaker power of 113 W.

The total content of free PC in *B. pendula* pollen extracts prepared by the orbital shaking method is close to the results obtained by ultrasound extraction. Processing of *B. pendula* with liquid nitrogen before extraction by orbital shaking did not affect the content of phenolic compounds in the extracts either (Table 2).

The total free PC content of *P. sylvestris* extracts detected by the orbital shaking method was 6% higher (Table 2) than that detected by the ultrasonic extraction method (Table 1). Processing of *P. sylvestris* pollen with liquid nitrogen did not affect the content of phenolic compounds. Conversely, extracts without liquid nitrogen had an 8% higher concentration of phenolic compounds compared to extracts treated with liquid nitrogen. In the extracts prepared by the orbital shaking method, the ratio of differences in free PC content between pollen types remained similar: 2.5 times more phenolic compounds were extracted from *B. pendula* pollen than from *P. sylvestris* pollen. In other stages of this research, the orbital shaking method was used for the extraction of free PC.

### 2.2. Free and Cell-Bound Phenolic Compounds, Flavonoid Content in Betula and Pinus Pollen Extracts

**Figure 1:**
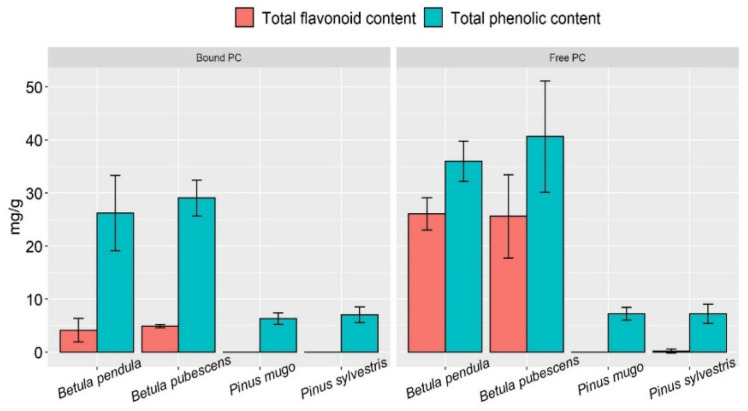
Total content of free PC, bound PC and flavonoids in *Betula* and *Pinus* pollen extracts.

**Figure 2:**
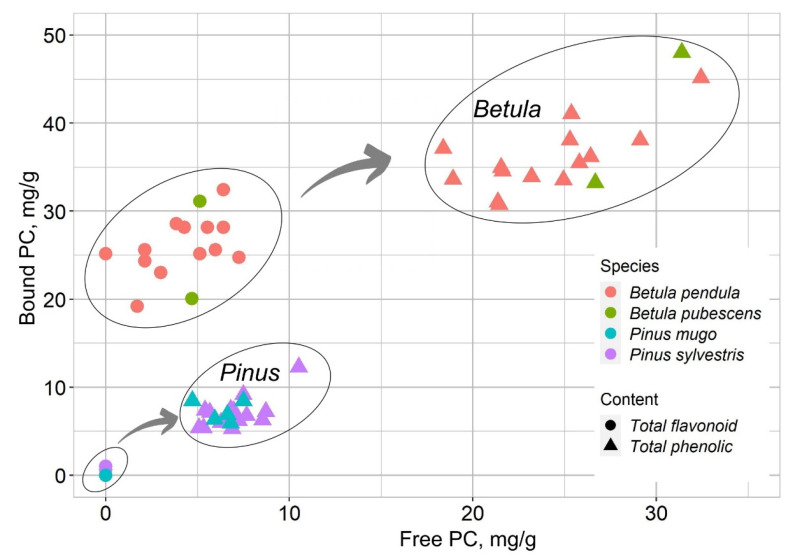
Total relative distribution of free PC, bound PC and flavonoids in *Betula* and *Pinus* pollen extracts.

For this study, we used pollen collected from different species of *Betula* (*B. pendula* and *B. pubescens*) and *Pinus* (*P. sylvestris* and *P. mugo*) plants. The concentration of free PC in *B. pendula* pollen extracts was on average 36.5 mg/g (Figure 1). About 60% of the total content of free PC consisted of flavonoids ~26.0 mg/g. The concentration in *B. pendula* bound PC extracts amounted to 25.0 mg/g on average and was 20% lower compared to free PC. Flavonoids were also found in bound PC extracts, but their content was unevenly distributed: in the range from 1.7 to 7.2 mg/g, and on average accounted for 16% of the total content of phenolic compounds. The average content of phenolic compounds in *B. pubescence* pollen was higher than that of *B. pendula* (Figure 1); however, the distribution of the data shows that the results are spread in the same range of concentrations (Figure 2).

In free PC and bound PC extracts, no significant differences were found between *Pinus* pollen species (Figure 1 and Figure 2). When evaluating the results of individual species, there were no differences in the content of free PC and bound PC in *P. sylvestris* pollen extracts. The average concentration of phenolic compounds was ~7.0 mg/g (Figure 1). *P. mugo* bound PC extracts contained less phenolic compounds compared to free PC extracts of the pollen of this species. Using the orbital shaking method, flavonoids were not detected in any of the *Pinus* pollen samples. Only solitary cases were found in the free PC extracts. All bound PC extracts contained flavonoids below the detection limit of the method.

### 2.3. Bioactivity of Betula and Pinus Pollen

In *Betula* pollen free PC extracts, the antioxidant activity according to DPPH• radical binding intensity was distributed between 60% and 80%. Free PC extracts showed about 20% higher antioxidant properties compared to bound PC extracts (Table 3).

**Figure 3:**
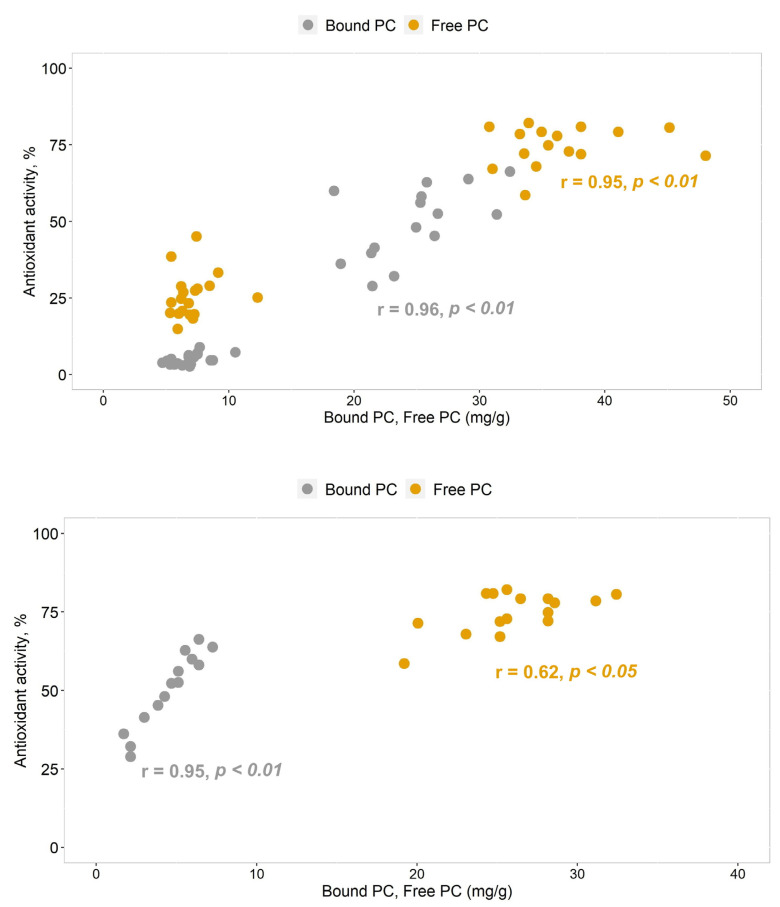
Correlations of total phenolic compounds (upper graph) and total flavonoids (bottom graph) content and antioxidant activity (according to DPPH•) in *Betula* free PC and bound PC extracts.

However, the compounds present in *Betula* bound PC extracts were also bioactive—i.e., statistically significant (*p* < 0.01) correlation coefficients of antioxidant activity were determined, both with free PC extracts and with phenolic compounds contained in bound PC extracts (Figure 3). It is likely that the bioactivity of compounds in bound PC extracts is influenced by flavonoids: as the content of flavonoids in bound PC extracts increased, the antioxidant activity also increased significantly (r = 0.95, *p* < 0.01).

In free PC extracts, phenolic compounds from the flavonoid group also had a significant impact on bioactivity (Figure 3), but the correlation coefficient was lower (r = 0.62, *p* < 0.05). When applying the ABTS• radical scavenging assay of wider polarity to the assessment of the bioactivity of phenolic compounds, similar results were obtained: the gap between the bioactivity of free PC and bound PC remained but did not exceed 10% (Table 3).

*Pinus* pollen extracts contain two times less bioactive compounds than *Betula* pollen. Only compounds present in free PC extracts showed higher antioxidant activity (Table 3). According to the DPPH• radical binding intensity, the antioxidant activity in free PC extracts was distributed between 17% and 40%. According to the ABTS• radical binding intensity, the antioxidant activity of *Pinus* free PC did not exceed or was lower than 20%. The bioactivity of *Pinus* compounds in bound PC extracts was also lower than the interval (20–80%) defining the limits of reliable antioxidant activity. Differences in the bioactivity of antioxidants in both *Betula* and *Pinus* pollen extracts were insignificant between species.

### 2.4. Chromatography of Betula and Pinus Pollen Phenolic Compounds

#### 2.4.1. High-Performance Thin-Layer Chromatography of *Betula* and *Pinus* Pollen Phenolic Compounds

**Figure 4:**
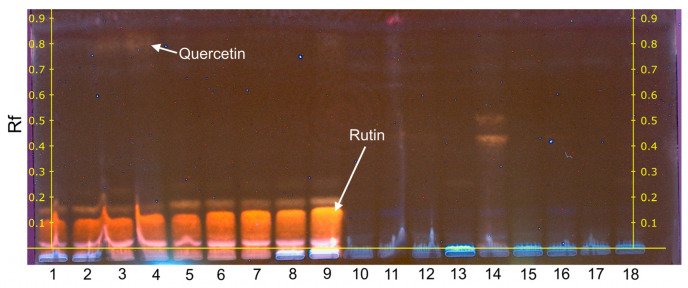
High-performance thin-layer chromatography of *Betula* and *Pinus* free PC extracts after derivatisation of DPPH• solution. Track indications: 1–9, *Betula* (injection volume 5 μL); 10–18, *Pinus* (injection volume 10 μL). Mobile phase consisted of acetone/chloroform/water (80:20:10 *v*/*v*/*v*).

**Figure 5:**
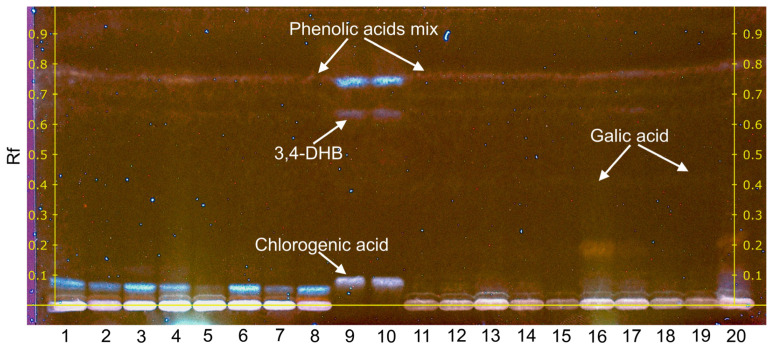
High-performance thin-layer chromatography of *Betula* and *Pinus* bound PC extracts after derivatisation of DPPH• solution. Track indications: 1–8, *Betula* (injection volume 5 μL); 9–10, phenolic acids mix (injection volume 2 µL); 11–20, *Pinus* (injection volume 10 μL). Mobile phase consisted of chloroform/ethyl acetate/acetone/formic acid (40:30:20:10 *v*/*v*/*v*/*v*).

Individual phenolic compounds present in *Betula* and *Pinus* pollen extracts were analysed by the high-performance thin-layer chromatography (HPTLC) method. HPTLC of standard substances of phenolic compounds was performed in parallel (Figure S1), and the results were compared with each other. The antioxidant activity of individual phenolic compounds was evaluated by processing HPTLC chromatograms of standard substances (Figure S2) and as a result of the derivatisation reaction with DPPH• solution. A part of the results is shown in Figure 4 and Figure 5, the other part is presented in the Supplementary Materials (Figures S1–S4).

The research has shown that the flavonoid rutin is dominant in all *Betula* free PC extracts and has a strong antioxidant effect (Figure 4, tracks 1–9). In samples 3 and 4, traces of quercetin are visible. After changing the composition of the mobile phase, the tracks of phenolic acids became distinct in the extracts of free PC (Figure S4). In *Betula* bound PC extracts, rutin was also detected (Figure S3), a strong chlorogenic acid signal was obtained (Figure 5, tracks 1–8) and traces of 3,4-dihydroxybenzoic acid were visible. TLC data of phenolic acid standards showed (Figure S1, Figure 5, tracks 9 and 10) that vanillic, trans-ferulic, p-coumaric, p-hydroxybenzoic, sinapic and syringic acids have the same retardation factor (Rf = 0.72). Thus, it was difficult to isolate other individual phenolic acids in the extracts. After derivatisation of phenolic acid standards with DPPH• radical (Rf = 0.72), gallic, trans-ferulic, chlorogenic and 3,4-dihydroxybenzoic acids were characterised by antioxidant activity (Figure S2).

In *Pinus* pollen free PC and bound PC extracts, rutin was present in single samples (Figure 4, track 15; Figure 5, tracks 16, 17 and 20), and its signal intensity was much lower compared to *Betula*. Traces of quercetin were also detected. In *Pinus* bound PC extracts, gallic acid (tracks 16, 20) and traces of 3,4-dihydroxybenzoic acid were determined, and a band of phenolic acids with the same retardation factor (Rf = 0.78) was visible (Figure 5).

#### 2.4.2. HPLC Analysis of *Betula* and *Pinus* Pollen Phenolic Compounds

**Figure 6:**
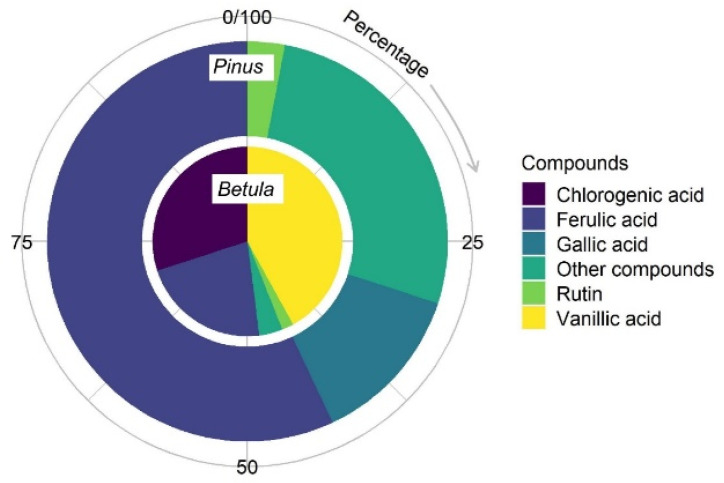
Relative distribution of phenolic compounds in bound PC extracts of *Betula* and *Pinus* pollen.

The variety of phenolic compounds in *Betula* and *Pinus* pollen extracts were analysed according to the UV spectra of standard substances: phenolic acids (gallic, vanillic, trans-ferulic, p-coumaric, p-hydroxybenzoic, 3,4-dihydroxybenzoic, sinapic and chlorogenic acids) and flavonoids (rutin and quercetin). The UV spectra and chromatogram of standard materials are presented in Table S1 and Figure S5. Chromatographic analysis of *Betula* pollen extracts showed that the phenolic acids in the extracts made up about 94% (Figure 6). Vanillic acid and chlorogenic acid were dominant, with 42% and 30%, respectively, and trans-ferulic acid constituted 22%. The amount of the flavonoid rutin was ~2%, and traces of 3,4-dihydroxybenzoic acid and quercetin were detected.

In *Pinus* pollen extracts, the absorption intensity of phenolic compounds was more than two times weaker than *Betula*. Seventy percent of the total amount of phenolic compounds identified was made up of two phenolic acids: trans-ferulic (57%) and gallic acid (13%). The amount of rutin in the extracts reached up to 3% according to the percentage distribution; however, the relative amount of rutin was lower than in *Betula* extracts. Other compounds accounted for 27% of the total amount of phenolic compounds identified.

## 3. Discussion

This research has revealed new characteristics of phenolic compounds in the pollen of angiosperms *Betula* and gymnosperms *Pinus*. The pollen wall of both gymnosperms and angiosperms is surrounded by a decay-resistant exine with its pores, biopolymer sporopollenin and inner layers [2,4,50,51]. Sporopollenin is characterised by stability, which allows pollen to remain intact for a long time in nature [52]. Phenolic compounds are important for the development and functions of pollen of anemophilous plants: they participate in the synthesis of sporopollenin and affect its stability [5,53], while bioactive properties help to protect the exine and intine [54]. Pollen phenolic compounds in the human diet are studied for both quantification and bioactivity determination [8,21,22,55]. Meanwhile, knowledge on the emission of phenolic compounds into the environment from naturally degrading pollen in ecosystems is very scarce. In our research, we have revealed new facts about the distribution of these compounds without breaking the pollen coat. We have to point out that our study focused on the release of phenolic compounds from pollen, creating extraction conditions closer to the natural environment.

Using two extraction methods, orbital shaking and ultrasound, we identified the property that the phenolic compounds are excreted with the cytoplasmic contents in a relatively short time. The same content of phenolic compounds was extracted from *Betula* pollen samples during the first 10 min of shaking as during 30 min in an ultrasound bath or during 16 h in an orbital shaking device. Research by [56] shows that *Betula* pollen bursts within 3 h when exposed to water, in addition it discharges cytoplasmic contents into the environment very quickly when stressed or exposed to moisture. A total of 6% more phenolic compounds were obtained from *Pinus* pollen free PC extracts using the orbital shaking method than using the ultrasound method. It is supposed that during longer exposure to methanol–water solution, more moisture was absorbed into *Pinus* pollen; therefore, the extraction of phenolic compounds using a 16-h duration of orbital shaking was more effective compared to that using 30 min ultrasonic shaking. According to [57], low-molecular-weight compounds containing carboxylic groups fill the main body of *Pinus sylvestris* pollen within 3 h.

The inner layers of the exine of *Pinus* pollen strictly control the permeability of carbohydrates to the central part, it is likely that there is also a serious barrier to the entry of water, alcohols, ampholytes and cations into the pollen [30], and compounds of higher molecular weight, including phenolic compounds, can enter only after rupture of the exine [8,57]. However, pollen stored for a long time loses some of its properties [58], the ability of pollen to release cytoplasmic contents to the environment weakens [39]. Forced release of *Pinus* pollen content into the environment requires stressful conditions: pressure and processing of pollen with inorganic acids, but even after *Pinus* pollen bursts, the spilled content is likely to remain enveloped in the intina layer [30].

To evaluate the strength of the pollen wall, the samples in our research were processed with liquid nitrogen. Analysing the effects of liquid nitrogen, we assume that cold has little effect on the release of phenolic compounds from pollen. The results of the research of both *Betula* and *Pinus* pollen showed that the use of liquid nitrogen did not increase the content of phenolic compounds in the extracts. A study [59] froze *Betula pendula*, *Betula pubescens* leaves and *Pinus sylvestris* needles with liquid nitrogen; however, freezing did not affect the content of phenolic compounds significantly either. The research [8] showed that superfine grinding wall disruption hardly changes the total soluble phenolic contents when 60 Co-irradiation increases.

The results obtained by our methods showed that the content of phenolic compounds in *Pinus* pollen was 2.5 times lower than *Betula*. In *Betula* pollen extracts, the difference between free PC and bond PC was 20%. We presume that this difference is determined by the emission of phenolic compounds from the cytoplasm. The low content of phenolic compounds in *Pinus* pollen extracts indicates that *Pinus* pollen phenolic compounds were not released into the extract from deeper layers but were released only from the pollen surface. This occurs because, not only in the deeper layers of the exine, but also on its surface, there are phenolic compounds that are not connected by covalent bonds [5]. Exine pores in *Pinus* pollen are small, but such pores are sufficient for fluids to enter the pollen interior [30]. It is likely that a sufficient amount of solvent was absorbed into the *Pinus* pollen in our research; however, the diffusion of phenolic compounds from the cytoplasm to the extract did not occur during the extraction process. The assumption is supported by the results, where the same concentration of ~7.0 mg/g phenolic compounds was found in both free PC and bound PC *Pinus* pollen extracts [30].

Our comparison of total phenolic compounds and total flavonoids between pollen extracts of different plant species of the same genus (*Betula* and *Pinus*) showed no obvious differences. Although *B. pendula* can be easily distinguished from *B. pubescens* morphologically, in many cases *B. pubescens* can be pollinated by *B. pendula* [60]. This may have implications for the phenolic compounds accumulated in pollen and the antioxidant activity of bioactive compounds. Research [59] shows that the contents of phenolic compounds accumulated in the leaves of *Betula pendula* and *Betula pubescens* are similar, while the concentrations of the same group of phenolic compounds in the needles of *Pinus sylvestris* are lower than in the leaves of *Betula*.

Plants accumulate a wide variety of phenolic compounds, which are divided into different classes based on the characteristics of their chemical structure [61]. The phenolic compounds of *Betula* and *Pinus* pollen are not widely studied; therefore, in our research we focused on phenolic acids of lower molecular weight soluble in water and polar organic solvents as well as on flavonoids widely distributed in plants, i.e., rutin and quercetin.

Analogous to the circumstances stated above regarding the content of phenolic compounds, the antioxidant activity of *Pinus* pollen was two times lower compared to *Betula*. The results of our research showed that rutin, chlorogenic acid and trans-ferulic acid were bioactive compounds in *Betula* and *Pinus* pollen extracts. Depending on the pollen morphotype, of all the phenolic compounds identified, phenolic acids accounted for 70–94% and rutin for 2–3%. We determined its strong antioxidant activity. Flavonoids are found in the plant cell as soluble free compounds and in a form bound to the cell wall [62]. They are important for protecting plants from pests and diseases, rutin is especially important for protecting plants from harmful UV radiation [63]. In the case of our research, the total flavonoid content of *Betula* free PC pollen extracts accounted for 60% of the total phenolic compounds. In addition to rutin, we detected traces of quercetin, but we did not determine any greater diversity of flavonoids in the extracts.

Chlorogenic acid, a compound with a strong antioxidant effect, was present in all *Betula* pollen samples, while it was found less frequently in *Pinus*. Chlorogenic acid, as one of the predominant phenolic acids, along with its derivatives are also found in other plant organs of the genus *Betula* [59,64]. Although [2] did not detect chlorogenic acid in *Betula* pendula pollen, they declared p-coumaric acid and ferulic acid to be bound to sporopollenin, and they identified p-coumaric acid as the main compound in the free PC fraction. Since chlorogenic acid loses its stability at low pH [44], the variety of more sensitive phenolic acids may vary depending on the conditions used for the extraction of phenolic compounds. According to [5], sporopollenin in *Pinus* pollen is bound by phenolic compounds: p-coumaric acid, p-hydroxybenzoic acid, vanillic acid, p-hydroxybenzaldehyde and ferulic acid.

In the examined samples, we found vanillic acid in *Betula* samples, while *Pinus* extracts contained gallic acid. Traces of 3,4-dihydroxybenzoic acid were detected in some of the *Betula* and *Pinus* pollen extracts we researched. One of the predominant acids in all samples without exception was trans-ferulic acid. This hydroxycinnamic acid is often found in plant cell walls [65]. According to the specific vibrational bands in infrared and FT-Raman spectra, derivatives of p-coumaric, ferulic and sinapic acids could be chemical imprints of the pollen cell wall [38].

The research added to the knowledge concerning phenolic compounds accumulated in the pollen of anemophilous plants and their bioactive properties which have had limited analysis so far. Indicators on the phenolic profiles of the pollen of *Betula* and *Pinus*, the most pollinating plants, and their antioxidant activity can be useful in modelling food chains in ecosystems, especially in water bodies, or parameters can be used to assess trophic potential. Pollen from *Betula* and *Pinus* is known to be dispersed in large quantities in the environment, water bodies and subsequently degraded by bacteria and aquatic microscopic fungi [18]. The research will be continued in order to obtain more information about the influence of phenolic compounds on trophic relationships in the ecosystem.

## 4. Materials and Methods

### 4.1. Chemical Substances

Standards of phenolic acids (gallic, vanillic, trans-ferulic, p-coumaric, p-hydroxybenzoic, 3,4-dihidroxybenzoic sinapic, chlorogenic and syringic acids) with purity ≥ 95–99%, rutin (≥94%) and quercetin (≥95%) were purchased from Merck (Darmstadt, Germany). Stock solutions were prepared by dissolving the solid compounds in methanol obtaining concentrations of 1.0 mg/mL. Methanol HPLC (99.9%), acetonitrile HPLC (99.9%), acetic acid (99.8%), trifluoroacetic acid (99%), 2,2-diphenyl-1-picrylhydrazyl radical (DPPH•), 2,2′-azino-bis (3-ethylbenothiazoline-6-sulphonic acid (ABTS) (98%) and 2N Folin–Ciocalteu reagent were purchased from Mersk (Darmstadt, Germany). Ethyl acetate, acetone and chloroform were purchased from J.T. Baker (Holland, The Netherlands).

### 4.2. Collection and Preparation of Betula and Pinus Pollen for Analysis

#### 4.2.1. Collection of Pollen In Situ

Catkins of *Betula pendula* and *Betula pubescens* with pollen were collected in late May and early June 2022. Mature strobils of *Pinus sylvestris* and *Pinus mugo* were collected in June 2022. All samples were collected from plants growing next to different water bodies in clean nature in Lithuania. The samples were isolated from each other throughout the study. Inflorescences of *Betula* and *Pinus* plants were brought to the laboratory and dried in a thermostat at a temperature of 35–40 °C (Memmert ULE 600, Schwabach, Germany) until the pollen began spreading. Then, the pollen was shaken out and separated from impurities by sieving. The pollen was stored in an ultra-low temperature freezer (Inova U102, New Brunswick, NJ, USA) at −70 °C until analysis.

#### 4.2.2. Principles of Analysis of *Betula* and *Pinus* Pollen Extracts

Section 2.1. describes *Betula pendula* and *Pinus sylvestris* pollen extracts used in the research. The efficiency of the release of free phenolic compounds (hereinafter referred to as free PC) into the water–methanol environment is evaluated.

Section 2.2 and Section 2.3 represent analyses of extracts of free PC and phenolic compounds bound to the cell wall (hereinafter referred to as bound PC) made from pollen of *Betula pendula*, *Betula pubescens*, *Pinus sylvestris* and *Pinus mugo*. The total content of phenolic compounds and flavonoids, and antioxidant activity were determined.

In Section 2.4.1, individual phenolic compounds and their bioactivity in *Betula* and *Pinus* pollen extracts were analysed by thin-layer chromatography (HPTLC) without separating the pollen into different species.

In Section 2.4.2, *Betula* and *Pinus* pollen extracts were used for the analysis of individual phenolic compounds by high-efficiency chromatography method, in which the largest total content of phenolic compounds was determined. The pollen was not divided into species.

### 4.3. Extraction of Phenolic Compounds from Betula and Pinus Pollen

#### 4.3.1. Extraction of Free Phenolic Compounds

**Figure 7:**
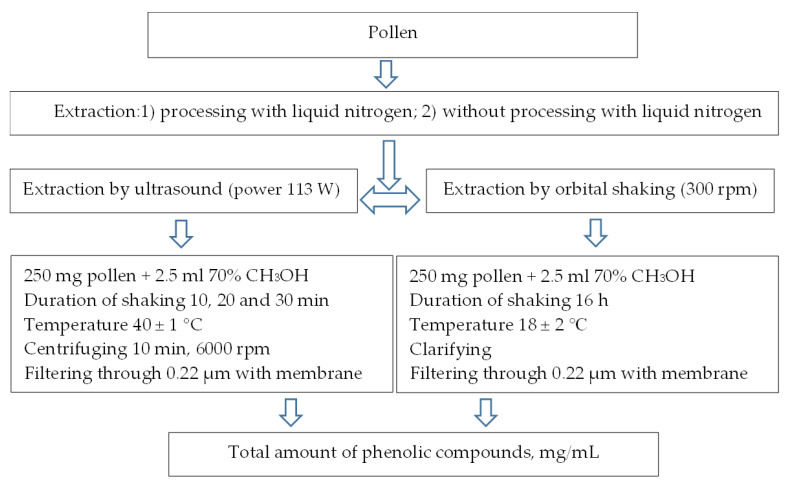
Scheme of methods applied for the extraction of free PC.

For the extraction of free PC from pollen, two methods were applied as well as the treatment of pollen with liquid nitrogen and a variant without treatment (Figure 7). An amount of ~1 g of *Betula pendula* and *Pinus sylvestris* pollen was frozen in liquid nitrogen and ground mechanically. Crushed pollen was poured with 70% methanol at a ratio of 1:10, incubated in an ultrasonic shaker (EMAG, Mörfelden-Walldorf, Germany) with different shaking durations and in an orbital shaking device (Heidolph Vibramax 100, Schwabach, Germany) with a constant duration of shaking. Depending on the method, the samples were then centrifuged (Eppendorf Centrifuge 5702, Hamburg, Germany), clarified, filtered and stored in a refrigerator (Snaigė RF59FB-P500270, Alytus, Lithuania) at +4 °C until analysis. Test results are provided in mg/mL.

Chemical analyses described in Section 2.4 were carried out using other extracts of free phenolic compounds. For their production, 100 mg of *Betula* and *Pinus* pollen dried to dry weight was added to 5 mL of 70% methanol and shaken in an orbital shaking device according to the scheme presented in Figure 7.

According to the results of the research on the extraction of phenolic compounds from *Betula* pollen, the use of an ultrasonic shaker with a short extraction time is sufficient to release the compounds into the solvent. However, in the case of *Pinus* pollen, better extraction of compounds was achieved by orbital shaking. To compare the distribution of phenolic compounds in the pollen of these different plants, orbital shaking was chosen, due to it being a method of longer duration and uniform extraction.

#### 4.3.2. Extraction of Bound-to-the-Cell-Wall Phenolic Compounds

Bound-to-the-cell-wall phenolic compound (bound PC) extracts were prepared according to [66] with a slight modification. An amount of 100 mg of *Betula* and *Pinus* pollen dried to a dry weight was poured into 2.5 mL of 0.1 M sodium hydroxide. Then it was shaken for one hour in an orbital shaking device (Heidolph Incubator 1000, Schwabach, Germany) at 40 °C, cooled to room temperature and acidified with 2 M of hydrochloric acid to pH 5–6. Up to 5 mL of methanol was added and shaken in an ultrasonic bath for 30 min. Then the sample was cooled, left to clarify, filtered through a 0.22 µm membrane filter (Chromafil ^®^Xtra PTFE-20/13, Düren, Germany) and stored until analysis at +4 °C.

### 4.4. Spectrophotometric Analysis of Phenolic Compounds of Betula and Pinus Pollen

#### 4.4.1. The Total Phenolic Compound Content

The total phenolic content of each extract was determined using the method, of the adapted version described in [22]. In the first stage, 100 μL of extracts or standard material was mixed with 7.9 mL of distilled deionised water; 0.5 mL of Folin–Ciocalteu reagent was added and incubated for 6 min at room temperature. Then 1.50 mL of 20% sodium carbonate was added. The absorbance of the solutions was measured at a wavelength of 760 nm, in a cuvette with a path length of 10 mm, with a UV/VIS spectrophotometer Genysis 10 UV (Thermo Spectronic, Rochester, NY, USA). The total content of phenolic compounds in pollen free PC and bound PC extracts was expressed in mg/g rutin equivalent, per dry weight (mg/g d.w.). The concentration limits of rutin were in the range of 0.05–1.5 mg/mL.

#### 4.4.2. The Total Content of Flavonoids

The total content of flavonoids was measured according to [67] with a slight modification. Amounts of 100 µL of extract and 300 µL of 5% sodium nitrate were added to 4 mL of distilled water. After 5 min, 300 µL of 10% aluminum chloride was added. The mixture was incubated for 6 min, then, 2 mL of 1 M sodium hydroxide was added to the mixture. The mixture was immediately diluted with 3.3 mL of distilled water (total volume 10 mL) and mixed well. Absorbance was measured with a spectrophotometer at a wavelength of 510 nm in a cuvette with a path length of 10 mm. A blank sample was prepared in parallel under the same conditions. Rutin was used to generate the calibration curve. The total content of flavonoids in *Betula* and *Pinus* free PC and bound PC extracts was expressed as rutin equivalents in mg per gram of sample dry weight (mg/g d.w.).

#### 4.4.3. Antioxidant Activity Using DPPH• and ABTS• Radical Scavenging Assays

The antioxidant activity of *Betula* and *Pinus* pollen extracts was determined by DPPH• and ABTS• radical scavenging assays. The method of [21] was applied for the determination of pollen extract antioxidant activity by DPPH•. An amount of 10 mg of DPPH• was dissolved in 250 mL of acetonitrile–methanol (1:1, *v*/*v*) solution and mixed with 250 mL of 0.1 M sodium acetate buffer, pH 5.5. The DPPH• solution was diluted to 0.50 ± 0.04 absorption units (a.u.) with acetonitrile–methanol–sodium acetate buffer solution. An amount of 77 µL of extract was added to 3000 µL of DPPH• solution, incubated at room temperature for 15 min in the dark. Absorbance was measured at a wavelength of 515 nm in a cuvette with a path length of 10 mm. A blank sample was prepared using the same procedure, replacing the extract with 70% methanol.

The ABTS• radical assay was carried out according to [68]. The stock 2 mM of ABTS solution was prepared by dissolving the reagent in 50 mL of phosphate buffer (PBS, pH 7.4). ABTS• was obtained by mixing 50 mL of the stock solution with 200 mL of 70 mM potassium persulfate. The mixture was left for 15–16 h in the dark at room temperature. A total of 10 µL of pollen extract was mixed with 3 mL of ABTS• solution with an absorbance intensity of 0.80 ± 0.02 a.u. Absorbance was measured in a 1 cm path length cuvette at room temperature 4 min later when the wavelength was 734 nm. PBS buffer solution was used as a blank sample.

Measurements were repeated at least twice. The antioxidant activity was calculated according to the following formula: activity (%) = (Ac − At/Ac) × 100, where At stands for the absorbance of the samples while Ac is the absorbance of the DPPH• or ABTS• blank sample. Absorbance measurements of extracts were compared with rutin calibration curves, which were performed as a positive control for both assays. The results were considered acceptable when the antioxidant activity was in the range from 20 to 80%.

### 4.5. Chromatographic Analysis of Phenolic Compounds of Betula and Pinus Pollen Extracts

#### 4.5.1. High-Performance Thin Layer Chromatography (HPTLC) Analysis

The HPTLC apparatus for high-performance thin layer chromatography was obtained from CAMAG (Muttenz, Switzerland). The system was equipped with a Linomat V Applicator, Visualizer and VisionCATS data processor (version 2.0). All analyses of standards mixtures and plant extracts were performed in a DS-L horizontal chamber obtained from Chromdes (Lublin, Poland). TLC plates with silica gel on aluminum foil background Kieselgel 60 F254 (Merck, Darmstadt, Germany) were applied. The plates were visualised under UV light (λ = 254 nm and λ = 366 nm). This equipment was used for qualitative analysis of standards solutions and plant extracts. A mobile phase consisting of chloroform: ethyl acetate: acetone: formic acid (40:30:20:10 *v*/*v*/*v*/*v*) was employed as the mobile phase for phenolic acids analyses, according to reference [69]. Flavonoids were analyzed using a solvent mixture, consisting of acetone: chloroform: water (80:20:10 *v*/*v*/*v*), according to reference [49]. The chromatograms on silica gel plates were processed for 30 min. The plates were covered with *Betula* and *Pinus* free PC and bound PC extracts. The mobile phase travelled a distance of 8.0 cm. The plates were dried at 40 °C and images were captured employing a Visualizer under a UV lamp. The detection was performed at visible light and at wavelengths λ = 254 nm and 365 nm. Moreover, the detection in the same conditions was performed after derivatisation by use of DPPH• solution for the spraying of the whole surface of the TLC plates. For the preparation of DPPH• solution (concentration level 10 mg/L), 1 mg of the pure substance was dissolved in 100 mL of methanol. All obtained chromatograms have been archived.

#### 4.5.2. High-Performance Liquid Chromatography (HPLC-DAD) Analysis

Extracts of *Betula* and *Pinus* pollen were analyzed by HPLC technique, using a system HPLC/Diode Array Detector (DAD) (Agilent 1260 Infinity, Agilent Technologies, Waldbronn, Germany) equipped with a reverse phase column ZORBAX Eclipse XDB (C18, 5 μm particle size, 150 × 4.6 mm, Agilent Technologies, Santa Clara, CA, USA). The column temperature was maintained at 20 °C.

Two gradient chromatographic methods were applied for the analysis of phenolic compounds.

Method 1. Phenolic compounds were separated in accordance with the method described by [70] with some modifications. The mobile phase was solvent A (100% methanol) and solvent B (10% acetonitrile and 2% of acetic acid in water). The program of elution was a linear gradient from 0 to 4 min 100% solvent B, 0% solvent A; 4–15 min 100% solvent A, 0% solvent B; 15–20 min 100% solvent A, 0% solvent B; 20–25 min 100% solvent B, 0% solvent A.

Method 2. The mobile phase was solvent A (0.01% TFA in water) and solvent B (0.01% TFA in ACN). The program of elution was a linear gradient from 0 to 15 min 5% solvent A, 95% solvent B; 18–15 min 5% solvent A, 95% solvent B; 18–21 min 85% solvent A, 15% solvent B; 21–23 min 85% solvent A, 15% solvent B.

The flow rate was 1.0 mL/min and column temperature was 20 °C. Sample volumes of 10 µL for standards, 20 µL for *Betula* and 40 µL for *Pinus* extracts were injected by auto-sampler.

Absorbance was measured in the wavelength range of 210–460 nm. Spectra of standard substances—gallic, vanillic, trans-ferulic, p-coumaric, p-hydroxybenzoic, 3,4-dihydroxybenzoic, sinapic and chlorogenic acids, as well as the flavonoids rutin and quercetin—were analysed in parallel (Table S1). The results are presented according to percentage distribution of phenolic compounds in pollen extracts.

### 4.6. Statistical Analysis

R software [71,72] was used for statistical data analysis. The variable means, standard errors and relative distribution of the total content of free PC and pollen bioactivity were calculated in pollen bound PC extracts. To evaluate the overall variation in the content of free, cell wall-bound phenolic compounds and flavonoids in pollen extracts, the data were visualised using the package ggplot2 [73], presenting the average values and calculating the standard error of mean. The overall relative distribution of free, cell wall-bound phenolic compounds and flavonoids in pollen extracts was evaluated using a scatter plot and grouping data by content and pollen type. Correlations of total phenolic compounds, total flavonoid content and antioxidant activity in free PC and bound PC extracts were determined by calculating Pearson correlation coefficients, and data dispersion was visualised using the package ggpubr [74].

## 5. Conclusions

It was evaluated that pollen released phenolic compounds into the methanol–water environment in a relatively short time using orbital shaking and ultrasound extraction methods. The results of the research of both *Betula* and *Pinus* pollen showed that the use of liquid nitrogen did not increase the content of phenolic compounds in the extracts. *Betula* pollen released 2.5 times more phenolic compounds than *Pinus* pollen.

When evaluating the contents of free PC and bond PC in pollen extracts, a 20% higher content of free PC was obtained in *Betula* pollen, whereas the content of phenolic compounds in *Pinus* pollen was the same (~7 mg/g) in both free PC and bound PC extracts. This can be explained that due to emission from the cytoplasm, *Betula* releases more phenolic compounds than *Pinus*.

Pollen bioactivity (*p* < 0.05) is related to the content of phenolic compounds and flavonoids in *Betula* free PC and bound PC, while in *Pinus*, only in free PC extracts. Antioxidant activity was characteristic of rutin, chlorogenic acid and trans-ferulic acid. Phenolic acids accounted for 70–94%, and the flavonoid rutin made up 2–3% of the total content of individual phenolic compounds identified in this research. One of the dominant phenolic acids was trans-ferulic acid in all *Betula* and *Pinus* samples. Specific compounds of *Betula* pollen extracts were vanillic and chlorogenic acids. It was found that *Pinus* extracts contained gallic acid.

The indicators on the phenolic profiles and antioxidant activity of the pollen of *Betula* and *Pinus*, the most pollinating plants, can be useful for modelling food chains in ecosystems, especially in water bodies. In order to obtain more information about the influence of phenolic compounds on trophic relationships in the ecosystem, research will be continued.

## Figures and Tables

**Table 1:** The content of free PC in pollen extracts isolated by ultrasound method.

Plant Species	Free PC, mg/mL According to the Extraction Duration					
	Without Liquid Nitrogen			With Liquid Nitrogen		
	10 min	20 min	30 min	10 min	20 min	30 min
*Betula* *pendula*	1.13 ± 0.03	1.07 ± 0.01	1.09 ± 0.03	1.09 ± 0.01	1.08 ± 0.05	1.09 ± 0.02
*Pinus sylvestris*	0.38 ± 0.01	0.40 ± 0.01	0.39 ± 0.01	0.37 ± 0.01	0.38 ± 0.01	0.38 ± 0.01

**Table 2:** The content of free PC in pollen extracts isolated by orbital shaking method.

Plant Species	Free PC, mg/mL, When Extraction Duration Is 16 h	
	Without Liquid Nitrogen	With Liquid Nitrogen
*Betula pendula*	1.09 ± 0.01	1.10 ± 0.01
*Pinus sylvestris*	0.46 ± 0.10	0.40 ± 0.02

**Table 3:** Bioactivity of *Betula* and *Pinus* pollen compounds.

Plant Species	DPPH^+^, %		ABTS^+^, %	
	Bound PC	Free PC	Bound PC	Free PC
*Betula pendula*	49.5 ± 11.1	75.5 ± 6.6	20.6 ± 4.0	37.2 ± 4.7
*Betula pubescens*	52.4 ± 11	75.2 ± 5.7	23.4 ± 6.0	31.9 ± 1.1
*Pinus sylvestris*	>LOD *	27.0 ± 9.7	>LOD	>LOD—20.0
*Pinus mugo*	>LOD	23.3 ± 5.5	>LOD	>LOD

* >LOD—below limit of detection.

## Data Availability

The data obtained in the experiment can be retrieved from the corresponding author upon reasonable request.

## Supplementary Materials

The following supporting information can be downloaded at: https://www.mdpi.com/article/10.3390/plants12020356/s1, Figure S1: High-performance thin layer chromatography (HPTLC) of phenolic acid and flavonoid standards. Figure S2. HPTLC of phenolic acid standards after derivatisation of DPPH• solution. Figure S3. HPTLC of *Betula* free PC and bound PC extracts after derivatisation of DPPH• solution. Figure S4. HPTLC of *Betula* and *Pinus* free PC extracts after derivatisation of DPPH• solution. Figure S5. HPLC-DAD spectrum of phenolic acids and flavonoid standards at 250 nm wavelength. Table S1. HPLC-DAD spectrum and parameters of phenolic acids and flavonoid standards.
