# Supplementary material for: Patterns of Phenolic Compounds in Betula and Pinus Pollen
Source: Plants (Basel). 2023 Jan 12;12(2):356. doi: 10.3390/plants12020356 (PMC9865354; doi:10.3390/plants12020356)
Supplement: Supplementary file 1 [file plants-12-00356-s001.zip › plants-2125089-supplementary.pdf]

SUPPLEMENT MATERIALL

Patterns of phenolic compounds in *Betula* and *Pinus* pollen

Ilona Kerienė, Ingrida Šaulienė, Laura Šukienė, Asta Judžentienė,
Magdalena Ligor, Bogusław Buszewski

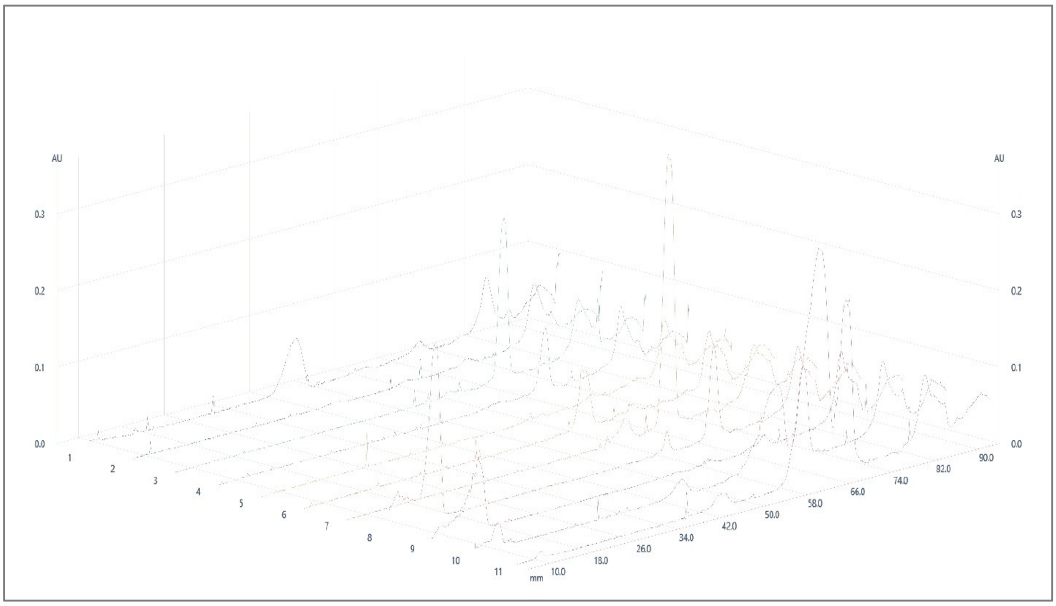

Figure S1. High performance thin layer chromatography (HPTLC) of phenolic acid and flavonoid standards. The sequence of standards indicates: 1 - gallic acid; 2 - vanillic acid; 3 - *trans*-ferulic acid; 4 - *p*-coumaric acid; 5 - *p*-hydroxybenzoic acid; 6 - 3,4-dihydroxybenzoic acid; 7 - sinapic acid; 8 - chlorogenic acid; 9 - syringic acid; 10 - rutin; 11 - quercetin. Injection volume 2 µl. The concentration of phenolic acids is 0.11 mg/mL (in methanol), rutin, quercetin - 0.05 mg/mL (in methanol). Mobile phase consisted of chloroform: ethyl acetate: acetone: formic acid (40:30:20:10 v/v).

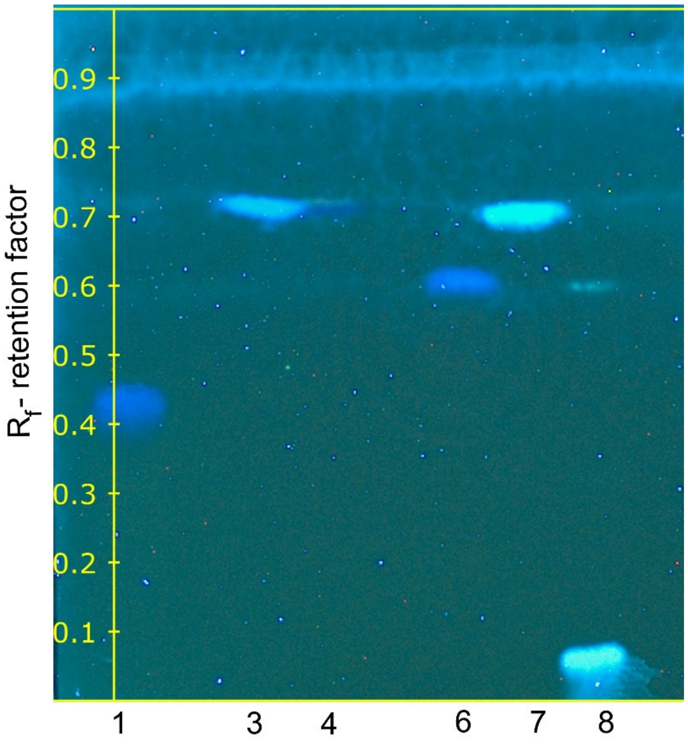

Figure S2. HPTLC of phenolic acid standards after derivatization of DPPH• solution. Glowing lines show the antioxidant activity of phenolic acids: 1 - gallic acid; 3 - *trans*-ferulic acid; 4 - *p*-coumaric acid; 6 - 3,4-dihydroxybenzoic acid; 8 - chlorogenic acid. Mobile phase consisted of chloroform: ethyl acetate: acetone: formic acid (40:30:20:10 v/v).

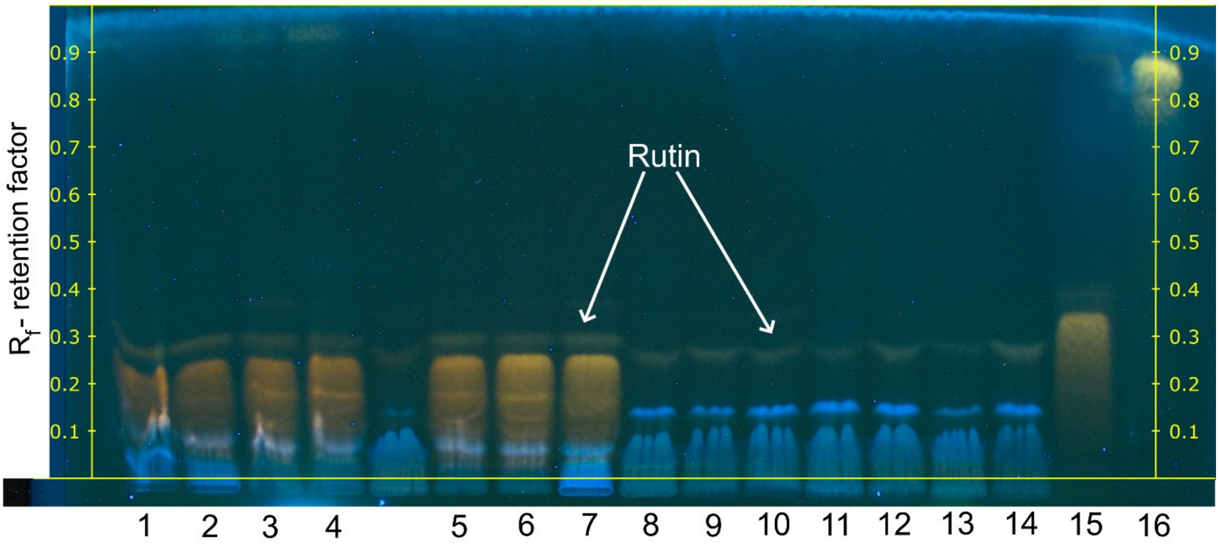

Figure S3. HPTLC of *Betula* free PC and bound PC extracts after derivatization of DPPH• solution. Track indications: free PC 1–7, bound PC 8–14. Standards of rutin - 15, quercetin - 16. Injection volume samples 10 µl; standards - 2 µl. Mobile phase consisted of acetone: chloroform: water (80:20:10 v/v).

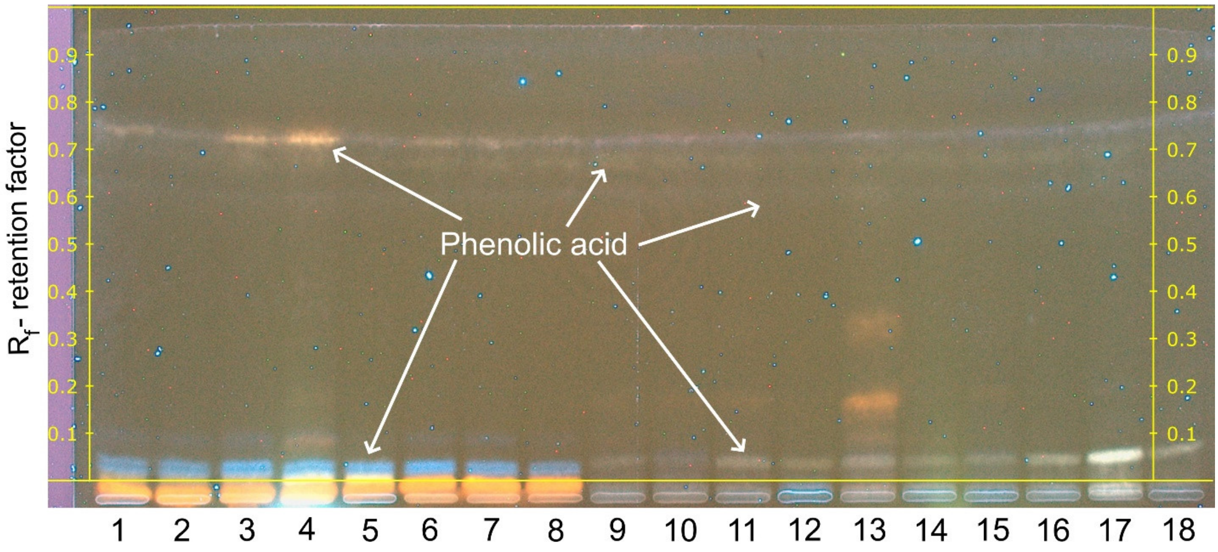

Figure S4. HPTLC of *Betula* and *Pinus* free PC extracts after derivatization of DPPH• solution. Track indications: 1–8 - *Betula* (injection volume 5 µl); 9–8 - *Pinus* (injection volume 10 µl). Mobile phase consisted of chloroform: ethyl acetate: acetone: formic acid (40:30:20:10 v/v).

Table S1. HPLC-DAD spectrum and parameters of phenolic acids and flavonoid standards

No	Phenolic compound	Retention time, min	Wavelength, nm	UV spectrum
1.	*trans*-Ferulic acid 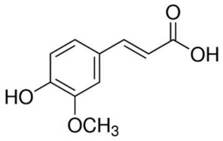	1.6	250, 260	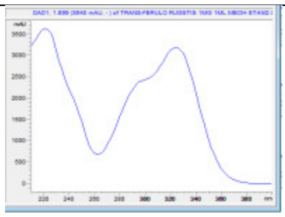
2.	Vanillic acid 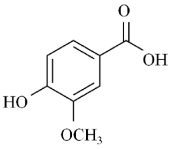	2.0	250, 260	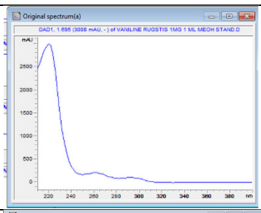
3.	Gallic acid 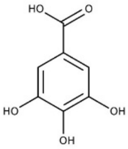	2.8	250, 260	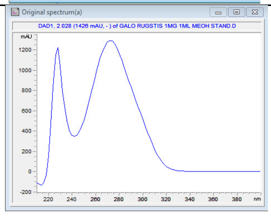
4.	3,4-Dihydroxybenzoic acid 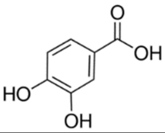	4.3	250, 260	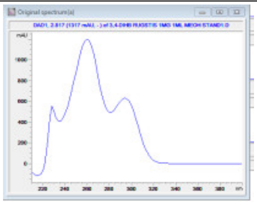
5.	*p*- Hydroxybenzoic acid 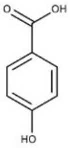	5.2	250, 260	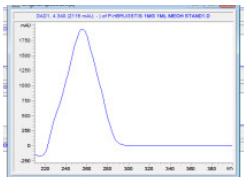
6.	*p*-Coumaric acid 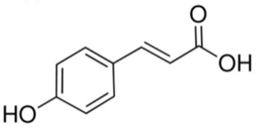	8.6	285, 310	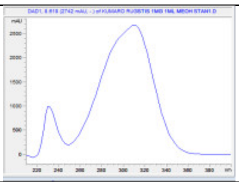
7.	Sinapic acid 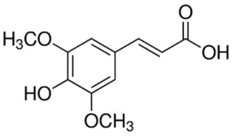	9.5	285, 310	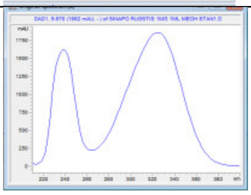

	Chlorogenic acid 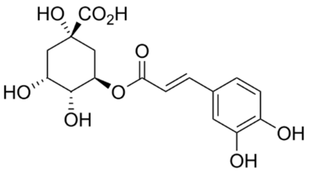	1.6	250, 260	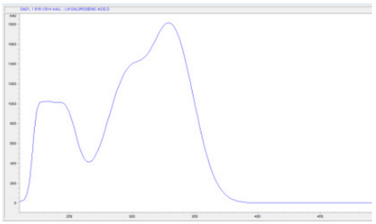
8.	Rutin 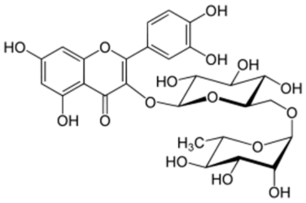	11.0	250, 260	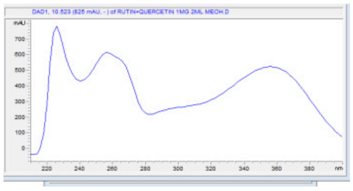
9.	Quercetin 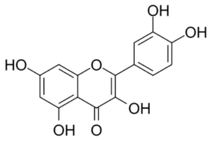	12.5	250, 260	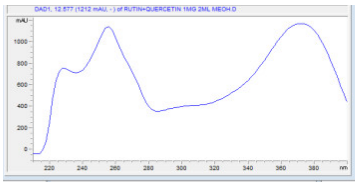

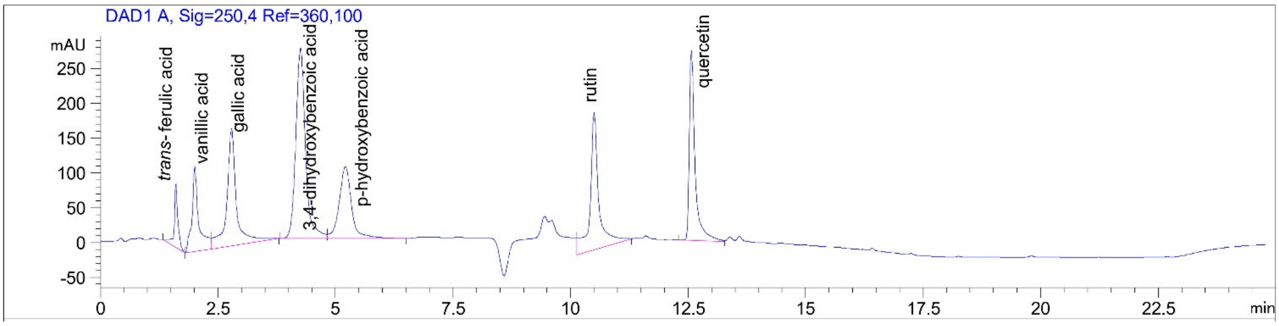

Figure S5. HPLC-DAD spectrum of phenolic acids and flavonoid standards at 250 nm wavelength. The standards concentration is 1 mg/mL, injection volume of 10 µl. The mobile phase is solvent A (100% methanol) and solvent B (10% acetonitrile and 2% acetic acid in water); the flow rate is 1 mL/min.
